# Role of Oxidative Stress and Inflammatory Cytokines (TNF-α and IL-6) in Acetic Acid-Induced Ulcerative Colitis in Rats: Ameliorated by *Otostegia fruticosa*

**DOI:** 10.3390/life11030195

**Published:** 2021-03-03

**Authors:** Mohd Nazam Ansari, Najeeb Ur Rehman, Aman Karim, Gamal A. Soliman, Majid A. Ganaie, Mohammad Raish, Abubaker M. Hamad

**Affiliations:** 1 Department of Pharmacology and Toxicology, College of Pharmacy, Prince Sattam Bin Abdulaziz University, Al-Kharj 11942, Saudi Arabia; g.soliman@psau.edu.sa; 2 Department of Biological Sciences, National University of Medical Sciences, Rawalpindi 46000, Pakistan; aman.karim@numspak.edu.pk; 3 Department of Pharmacognosy, School of Pharmacy, College of Health Sciences, Mekelle University, Mekelle 1871, Ethiopia; 4 Department of Pharmacology, College of Dentistry and Pharmacy, Buraydah Colleges, Buraydah 51452, Saudi Arabia; majid.ahmad@bpc.edu.sa; 5 Department of Pharmaceutics, College of Pharmacy, King Saud University, Riyadh 11451, Saudi Arabia; mraish@ksu.edu.sa; 6 Department of Basic Sciences, Preparatory Year Deanship, Prince Sattam Bin Abdulaziz University, Al-Kharj 11942, Saudi Arabia; a.hamad@psau.edu.sa; 7 Department of Histopathology and Cytopathology, Faculty of Medical Laboratory Sciences, University of Gezira, Wad Madani 21111, Sudan

**Keywords:** acetic acid, Ulcerative colitis, *Otostegia fruticosa*, IL-6, oxidative stress

## Abstract

Ulcerative colitis (UC) is an inflammatory bowel disease (IBD) that causes irritation, inflammation, and ulceration in the linings of the colon and rectum. *Otostegia fruticosa* is traditionally used to treat various disorders in different parts of the Middle East and sub-Saharan Africa. In the present study, we evaluated the ameliorative effects of crude leaves extract of *O. fruticosa* (OF.Cr) on acetic acid (AA)-induced UC model in Wistar albino rats. Wistar rats were administered orally with either vehicle (10 mL/kg), OF.Cr (200 and 400 mg/kg), or prednisolone (2 mg/kg) once a day for 6 days. On day 6, UC was induced in rats by intrarectal administration of a single dose of 5% AA (1.0 mL). Disease activity index (DAI) was recorded after one day of colitis induction by assessing the symptoms of colitis and then the rats were euthanized by cervical dislocation, and colon tissues were isolated for the histopathological examination and biochemical analysis of oxidative stress parameters and cytokines (Interleukin-6 and Tumor Necrosis Factor-α). OF.Cr pretreatment exhibits significant prevention against UC, as confirmed by a significant decrease of DAI, colonic ulceration, and reduced inflammatory score as compared to the AA-induced colitis rats. Depletion of total glutathione (GSH) levels and catalase (CAT) activities in the colitis group was significantly restored in the OF.Cr treated groups, while increased lipid peroxidation in the colon tissues was significantly reduced. OF.Cr prevented the activation of the IL-6 and TNF-α pathways in the colonic tissues, which were clearly observed by the decreased levels of IL-6 and TNF-α in the OF.Cr treated animals. Hence, OF.Cr could be developed in the future for the treatment of UC.

## 1. Introduction

Ulcerative colitis (UC) is a non-specific and relapsing inflammatory bowel disease (IBD) that causes irritation, inflammation, and ulcers in the innermost lining of the colon and rectum. UC is characterized by pathological mucosal damage and ulceration. Various factors, such as hereditary, lifestyle, immune disturbances, environmental factors, and intestinal dysbiosis, may contribute to UC [1,2]. UC exhibits chronic diarrhea (often with pus or blood), abdominal pain, cramps, and rectal bleeding. Remarkably, its prolonged infection generally results in serious problems, even cancer of the colon and rectum [3]. Previous literature has reported that UC is more prevalent in developed countries; however, in recent years it has been observed that the frequency appears to be more in Asian countries but has still not achieved the same prevalence as it has in countries of the western hemisphere [4].

Because of the increased similarity between experimentally-induced UC in animals and UC in patients, the experimentally-induced UC in different animal models has become more popular in research to study the pathological mechanism of the disease and also for the study of new drugs for their promising role in the treatment of UC [5]. Among the various pathological outcomes associated with UC, an enhanced release of inflammatory mediators, such as prostaglandins and leukotrienes, is more common. Previous studies confirmed the role of prostaglandin E2 (PGE2), thromboxane A2, prostacyclin, and leukotriene B4 (LTB4) in colonic inflammation [6]. Altered colonic environment, unusual mucosal glycosaminoglycan, reduced oxidation of short-chain fatty acids, enhanced intestinal permeability, and sulfide production are key factors in the pathogenesis of IBD [7]. A study reported by Yasukawa et al. [8] showed the imbalance between oxidative stress and antioxidant protective system in experimentally induced UC in common laboratory animals.

For the management of IBD including UC, the most commonly used drugs are sulphasalazine, 5-aminosalicylic acid, corticosteroids, azathioprine, methotrexate, 6 mercaptopurine, calcineurin inhibitors (cyclosporine and tacrolimus), and anti-TNF-α antibodies (infliximab, adalimumab, and certolizumab), which aim to control inflammation of the mucosa, complications, and disease relapse [9,10]. Selection of the drugs is based on the extent and severity of the disease, response to current or prior treatment, and complications [11]. Regrettably, the available drugs are not much effective as they need prolonged use and also result in drug intolerance, adverse drug reactions, and allergic reactions [12]. Therefore, natural products and plant-based drugs could be an option for the treatment of UC as, in traditional medicine, these are being paid much courtesy because of minimal side effects, their inexpensiveness, and the fact that most of the world’s populations still trust them [13]. Numerous studies reported the effect of several natural products in UC including *Aloe vera* [14], *Gingko biloba* [15], Liquorice [16], fenugreek [17], Curcumin [18], *Zingiber officinale* [19], and Green tea [20].

The genus Otostegia contains about 33 species under the family Lamiaceae. *O. fruticosa* (Forssk.) Schweinf. ex Penzig is extensively spread in Ethiopia, Sudan, Cameroon, Saudi Arabia, Yemen, and Palestine. Previous studies reported the traditional use of *O. fruticosa* leaves in various disorders such as airway disorders, tonsillitis, and acute fever [21,22]. The smoke of burned leaves has been used for stomach ache [23]. Andemariam [24] reported the uses of aerial parts of the plant in arthritis, tonsillitis, and gynecological problems. According to Rahman et al., [25], the flowering branches of this plant are used traditionally to treat sunstroke. In the previous study, we reported the bronchodilatory effect of the *O. fruticosa* crude extract [26].

The anti-inflammatory and antidiarrheal effect of *O. fruticosa* has been reported in earlier studies [27,28]. Pharmacological investigation on different extracts of *O. fruticosa* claims antimicrobial and antioxidant activities [29]. Recently, Al-Jumayi has reported ameliorative effect of *O. fruticosa* essential oil against gentamicin-induced nephrotoxicity [30]. Various traditional uses have been reported for *O. fruticosa*, but no scientific data are available on the potential of the plant in UC. Thus, on the basis of earlier reports, the present experiment planned to explore the possible ameliorative effect of the *O. fruticose* crude extract against acetic acid (AA)-induced UC on a murine model by evaluating the oxidative stress and the macroscopic and microscopic parameters. The underlying mechanisms were also determined by assessing the antioxidant potential of the extract and/or decreased levels of pro-inflammatory cytokines in colon tissues.

## 2. Materials and Methods

### 2.1. Chemicals and Reagents

Prednisolone was procured from Sigma–Aldrich, St. Louis, MO, USA, and all other chemicals used in the study were of analytical grade.

### 2.2. Plant Collection and Extraction

The leaves of *O. fruticosa*, collected from Wukro Kilteawlaelo, located at a distance of 42 Km East of Mekelle, Northern Ethiopia, were identified by Mr. Shamble Alemu. The plant sample with a voucher specimen (001) was submitted for recording at the National Herbarium, College of Natural and Computational Science, Addis Ababa University.

The shad dried leaves of *O. fruticosa* were ground into coarse powder, which was then soaked in 70% ethanol (*w*/*v*) with shaking by orbital shaker (130 rpm) for proper mixing for three days. The filtrate was passed through a Whatman filter paper grade 1, and the maceration process was repeated thrice. All three filtrates were combined and then dried in a rotary evaporator at 40 °C. The final semi-solid crude extract of *O. fruticosa* (OF.Cr) was stored in the refrigerator for further use.

### 2.3. Animals

Approximately 180–200 g body weight, male Wistar albino rats were selected in this study. The available laboratory animal facility, located at the College of Pharmacy, Prince Sattam bin Abdulaziz University (PSAU), Saudi Arabia, was used to obtain and house the animals with standard temperature (22 ± 1 °C), relative humidity (55 ± 5%), and 12 h/12 h light/dark cycle. All animals were provided a standard pellet diet with filtered drinking water. Two increasing doses of OF.Cr and a positive control drug (prednisolone) were administered orally (PO). Scientific guidelines, as detailed in the Institute of Laboratory Animal Resources, Commission on Life Sciences, National Research Council [31], were followed.

### 2.4. Acute Toxicity Study

The detailed guidelines reported in OECD-423 were followed for calculating the maximum tolerated dose using an acute toxicity test [32]. Briefly, overnight fasted albino rats (n = 6) were distributed into two groups in a random fashion. One group of animals was exposed for oral administration of OF.Cr (2 g/kg), while the second group, labeled as a vehicle control group, received only vehicle (3% *v*/*v* Tween 80 dissolved in distilled water), with a dose of 10 mL/kg. Both groups of rats were maintained at similar housing conditions, and each individual rat was continuously observed for 4 h for any sign of toxicityor deaths at every 15 intervals, then every half hour for the next 6 h, and then daily for two days (48 h). Because no mortality was observed at 2 g/kg, the dose of OF.Cr was increased to 4 g/kg and the treated rats were further observed for the next 48 h.

### 2.5. Study Design

**Figure 1:**
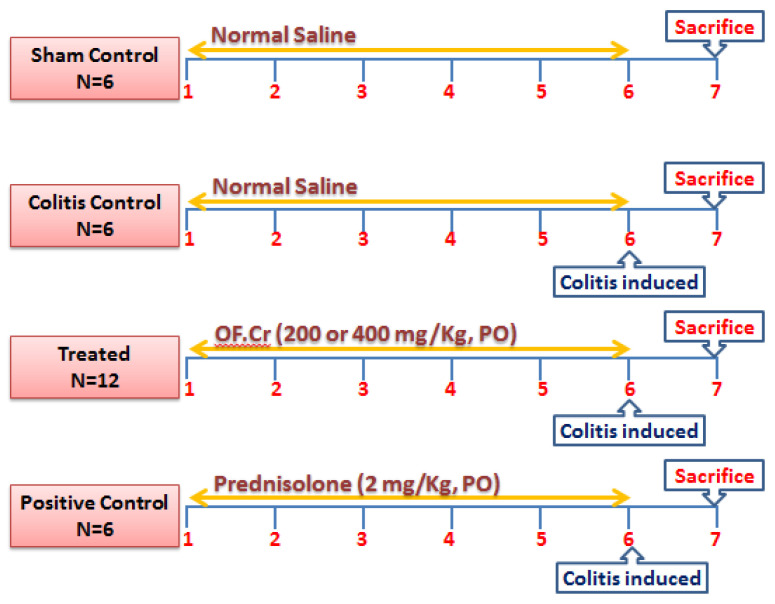
A graphical scheme of the experiment.

Thirty male healthy rats were randomly divided into 5 groups of rats (n = 6). Group 1 (Sham) and group 2 (colitis control) were medicated with the vehicle. Animals of groups 3 and 4 were treated with *O. fruticosa* extract at doses of 200 or 400 mg/kg, respectively. Group 5 (positive control) animals were administered prednisolone (2 mg/kg). Normal saline, *O. fruticosa* extract, and prednisolone were administered daily for 6 days, and the last dose was given 120 min earlier than the colitis induction. A graphical scheme of the experiment is shown in Figure 1.

### 2.6. Induction of Colitis

Intrarectal (IR) administration of 5% acetic acid (AA) is an easy and reproducible experimental model for colitis induction that manifests the same features of human UC [33]. Rats were fasted for 16 h with free access to water and were then lightly anesthetized with pentobarbital sodium (30 mg/kg, IP).

Rectal administration of 1.0 mL of AA (5% *v*/*v*) was used to induce ulcerative colitis in all the groups except those labeled as the sham group. For this purpose, a polyethylene catheter was selected and inserted into the rectum of each fasted rat such that it enters up to 8 cm into the anus. Each rat was held for 45 s in an upside-down position to avoid any rectal emission of the AA. Further, to neutralize the severity of the AA solution, each rat rectum was washed with saline (0.5 mL).

Following 24 h of colitis induction and euthanizing these rats, the parameter of disease activity index (DAI) was scored for each rat. Their colon tissues were isolated for the assessment of any macroscopic and microscopic changes while biochemical estimations of oxidative stress-related indicators (malondialdehyde (MDA), glutathione (GSH), and catalase (CAT)) and cytokines (IL-6 and TNFα) were determined.

### 2.7. Assessment of Disease Activity Index (DAI)

The clinical severity of colitis was evaluated by observing the body weight, stool uniformity, and presence/absence of blood in the rectum between the time of administration of AA and euthanasia, in a blinded approach [34]. To record DAI, the average of the above-mentioned parameters was considered, as detailed in Table S1.

### 2.8. Assessment of Macroscopic Damage

The indicators of inflammation (edema) were recorded by assessing the macroscopic damage, which was calculated as follows; firstly, after isolation of spleen and 8 cm of colon tissue proximal to the anus from respective treatment group animals, their wet weight (g/cm) was measured using an electronic balance, while the normal scale was used to measure their lengths (cm).

Secondly, each isolated colon tissue was opened longitudinally following their mesenteric lines, and their percentage affected area was measured in terms of hyperemia with or without lesions. Thirdly, the ulcer index was calculated by summing up the score of ulcer lesion and ulcer area [35]. Fourthly, the colon tissues lesion score of the ulcer [36] indicates the qualitative nature of the ulcer by the following increasing score numbers:

No macroscopic changes were labeled as zero (0), mucosal erythema was counted as one (1), mild mucosal edema with slight bleeding or slight erosion was scored two (2), moderate edema, bleeding ulcers, or erosions were scored as three (3), whereas a score of four (4) indicates extensive ulceration, erosions, edema, and tissue necrosis.

Graph paper was used to quantify the area of ulcer lesion of the colonic tissues by placing the selected colon section on the graph paper where each small box area was equal to 1 mm^2^. The ulcer index was calculated by using the following equation;

Ulcer Index (UI) = Lesion Score + Area of Ulcer Lesion

### 2.9. Preparation of Tissue Homogenate

Following a cervical dislocation, colon tissues were excised from all the respective treatment groups of animals and immediately perfused with ice-cold normal saline. After manual mincing, each tissue was further processed for the required homogenization using a Potter–Elvehjem homogenizer in chilled phosphate buffer (0.1 M; pH 7.4) as a medium. The obtained tissue homogenates were centrifuged at 700× *g* and 4 °C for 10 min to separate nuclear debris. The supernatants were obtained and were exposed to one more centrifugation at 9000× *g* and 4 °C for 20 min to isolate a post-mitochondrial supernatant (PMS) [37].

### 2.10. Assessment of Lipid Peroxidation Activity

For the assessment of lipid peroxidation, levels of malondialdehyde (MDA) were measured in all treatment groups of animals [38]. Homogenate (0.25 mL) was incubated at 37 °C in a metabolic shaker for 1 h. After 1 h of incubation, 0.5 mL of 5% (*w*/*v*) chilled TCA and 0.5 mL of 0.67% thiobarbituric acid was added, followed by centrifugation (1000× *g*, 15 min). Thereafter, supernatant was placed in a boiling water bath for 10 min. The absorbance of pink color developed was measured at 535 nm.

### 2.11. Assessment of Antioxidant Levels

Antioxidants were assessed in the PMS of colon homogenate of all the experimental rats by measuring tissue total glutathione (GSH) and catalase enzyme activity (CAT) levels.

For GSH, the method of Jollow et al. [39] was followed. One mL PMS was precipitated with 1.0 mL of sulfosalicylic acid (4%). The samples were incubated at 4 °C for 1 h followed by centrifugation (1200 g, 15 min, 4 °C). The assay mixture contained 0.1 mL supernatant, 1.7 mL phosphate buffer (0.1 M, pH 7.4), and 0.2 mL dithio-bis-2-nitrobenzoic acid (DTNB) (0.4% in phosphate buffer, 0.1 M, pH 7.4) in a total volume of 2.0 mL. Briefly, the absorbance of the samples was recorded within 5 min at 412 nm after the addition of DTNB to the reaction mixtures.

The earlier reported method was followed stepwise to estimate the CAT levels in the isolated colonic tissue of each rat [40]. The reaction mixture consisted of 1.95mL of phosphate buffer (0.1 M, pH 7.4), 1.0 mL of hydrogen peroxide (0.019 M), and 0.05 mL of PMS in a final volume of 3ml. Changes in absorbance were recorded at 240 nm every min for 5 min.

### 2.12. Assessment of Cytokine Levels (IL-6 and TNFα)

The secretory levels of cytokines in the PMS of the colon tissue of all rats were assessed. The PMS was assayed for the quantitative measurement of mucosal IL-6 and TNF-α with their respective enzyme linked immunosorbent assay (ELISA) kits, as instructed by the manufacturer (eBIOSCIENCE, Bender MedSystems GmbH, Wien, Austria). Inter and intra-assay coefficients of variation (CV) of IL-6 was 8.9% and 5.0%, respectively, while inter and intra-assay CV for *TNFα* was 5.7% and 6.5%, respectively.

### 2.13. Microscopic Assessment of UC

For the possible protective effect of the *O. fruticose* on microscopic examination of colonic tissues of rats exposed to AA, the intensity of colonic tissues damage was assessed by staining with standard methods by using hematoxylin and eosin (H & E) stains. The depletion and/or retention of goblet cells were examined by using periodic acid Schiff’s (PAS) staining.

After excision, the obtained colonic tissue samples approximately 3-5cm proximal to anus were transferred immediately to neutral buffered formalin and stored for further use. Each tissue sample was initially exposed to processing in an automatic tissue processing machine (ASP300s, Leica Biosystems, IL, USA) and embedded in paraffin wax. A section with a thickness of 5 µM was prepared using a rotary microtome (SHUR/Cut 4500, TBS, NC, USA). From each colonic tissue, two sections were selected and processed; one was stained by H&E as a general staining, while another was stained with PAS stain for neutral mucin (Kiela et al., 2005). For the quality characterization of any histological changes, all selected tissues were examined using an Olympus BX 52 microscope. A histopathologist, who was unaware of the treatments, observed and characterized the histological scores. Pictures were obtained using a digital camera (Olympus DP21) attached with a microscope.

To score the microscopic damage and to evaluate the possible protective effects of *O. fruticose* and respective controls, scoring criteria were classified into three types, as detailed: Neurath (score 0–4) [41] and Kiela (score 0–3) [42], while the loss of goblet cells was assessed using mucin staining as the base to quantify their loss [43].

### 2.14. Statistical Analysis

All findings are presented as the median (Interquartile range = IQR). One-way analysis of variance (ANOVA) followed by post hoc Tukey’s test was used to compare different treatment groups with disease group animals. The results were also substantiated by nonparametric Kruskal–Wallis test followed by Dunn’s test, for the comparison of microscopical changes. Statistical significance was considered if *p* < 0.05. The analysis was performed using GraphPad Software, San Diego, CA, USA (version 4).

## 3. Results

### 3.1. Effect of O. fruticosa Extract on the Disease Activity Index

**Figure 2:**
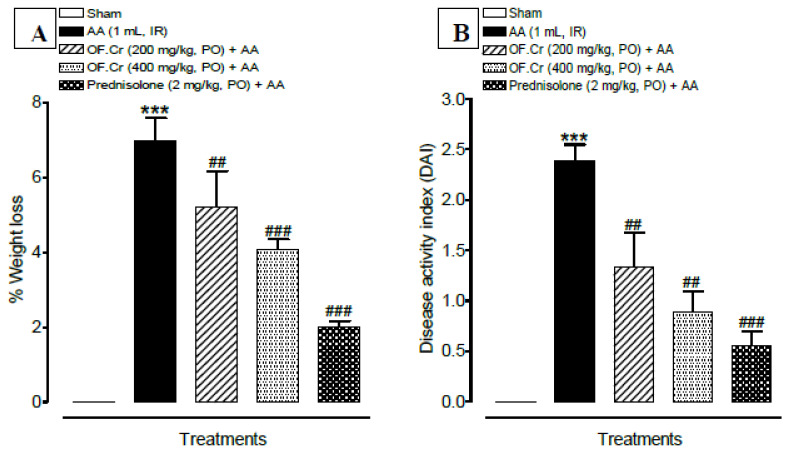
Pretreatment with OF.Cr improved percentage of (**A**) weight loss and (**B**) disease activity index (DAI) in Wistar albino rats induced with AA colitis. Data expressed as median (IQR) (n = 6). One way ANOVA was used for statistical analysis followed by Tukey’s post-test. *** *p* < 0.001 compared with sham control, ^##^ *p* < 0.01, ^###^ *p* < 0.001 compared with AA alone. OF.Cr = *Otostegia fruticosa* crude extract. AA = acetic acid; PO = orally; IR = intrarectally.

The extent and severity of the colitis induced by acetic acid administration were evaluated in all of the animals. AA-induced colitis rats exhibited a marked decrease in body weight, as shown in Figure 2A. However, OF.Cr and prednisolone pre-treatment in colitis rats resulted in a significant decrease in body weight loss as compared to the colitis control rats.

The DAI score is used as a significant indicator to assess the colonic injury and assessed from signs such as decreased bodyweight, diarrhea, and blood stools. The lower DAI score is considered as the animal is closer to the normal physiological state. In the present study, AA-induced colitis rats exhibited symptoms of UC, as indicated by diarrhea and bloody stools. AA-induced colitis rats exhibited significantly (*p* < 0.001) increased DAI scores when compared to the sham control. However, OF.Cr administered rats showed significantly (*p* < 0.01) reduced DAI scores at both doses (Figure 2B).

### 3.2. Effect of O. fruticosa Extract on Macroscopic Anatomy

**Figure 3:**
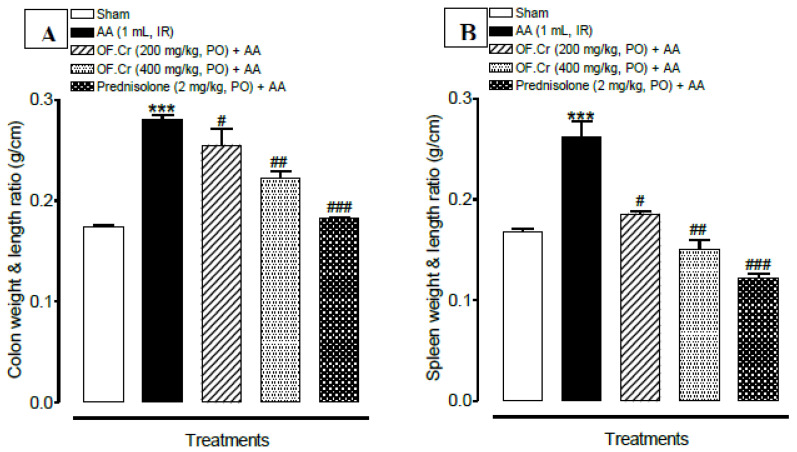
Effect of *Otostegia fruticosa* on (**A**) colon weight and length ration and (**B**) spleen weight and length ration) in AA-induced colitis rats. Data expressed as median (IQR) (n = 6). One way ANOVA was used for statistical analysis (ANOVA *p*-value < 0.0001) followed by Tukey’s post-test. *** *p* < 0.001 compared with sham control, ^#^ *p* < 0.05, ^##^ *p* < 0.01, ^###^ *p* < 0.001 compared with AA alone. OF.Cr = *Otostegia fruticosa* crude extract. AA = acetic acid; PO = orally; IR = intrarectally.

The ratio of wet weight and length of rat colon and spleen were determined for macroscopic examination. AA-induced colitis rats were reported with an increased weight/length ratio of the colon and spleen. OF.Cr and prednisolone pretreatment resulted in a comparatively smaller ratio as compared to colitis animals, which indicates a decrease in inflammation (Figure 3A,B).

**Figure 4:**
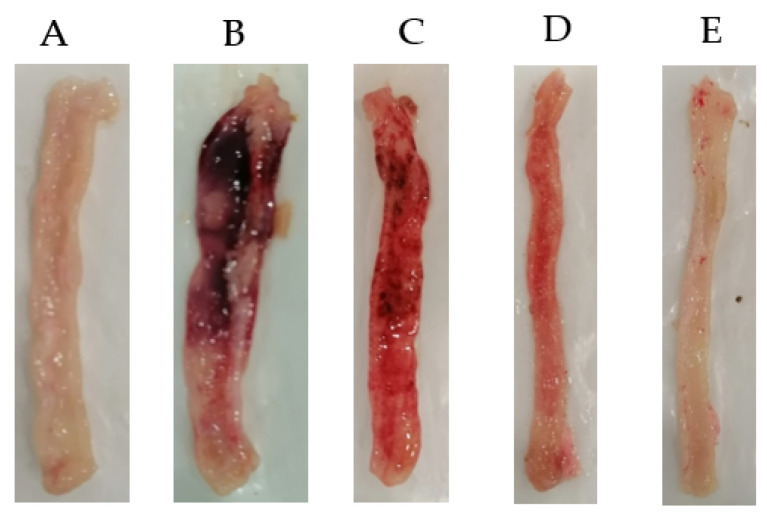
Effect of *Otostegia fruticosa* crude extract on the gross anatomy of colons against acetic acid-induced colitis in Wistar albino rats. (**A**) Sham control rat, (**B**) Colitis control (AA) rats, (**C**) *Otostegia fruticosa* crude extract (200 mg/kg) + colitis, (**D**) *Otostegia fruticosa* crude extract (400 mg/kg) + colitis and (**E**) Prednisolone (2 mg/kg) + colitis.

Colon tissues of the sham control group exhibited the typical intact structure with no lesions or hyperemia (Figure 4A). The colonic specimens of the colitis control (AA) rats appeared ulcerated, oedematous, and hemorrhagic (Figure 4B). Pretreatment of rats with OF.Cr at 200 and 400 mg/kg and prednisolone significantly restore these macroscopic injuries produced by the AA (Figure 4C–E).

**Figure 5:**
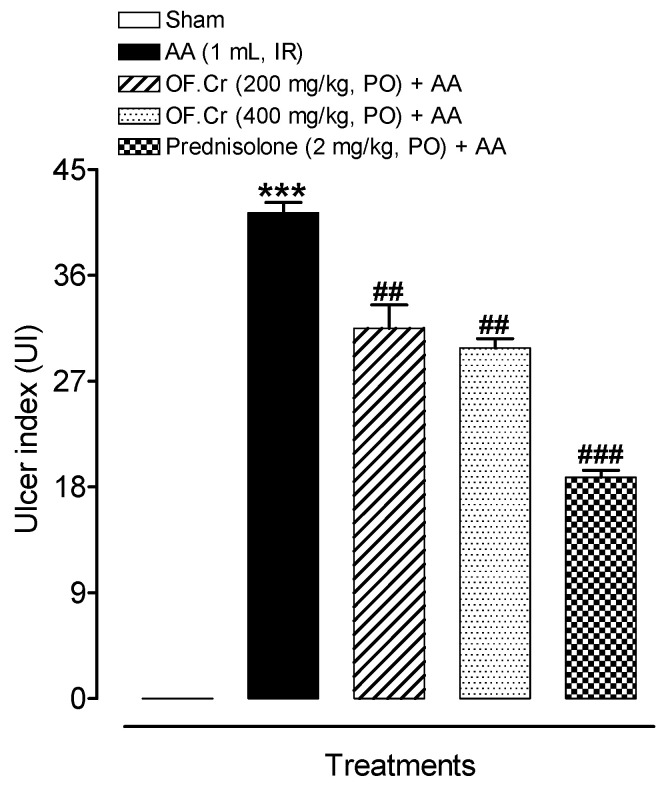
Effect of *Otostegia fruticosa* on ulcer index in AA-induced colitis rats. Data expressed as median (IQR) (n = 6). One-way ANOVA was used for statistical analysis followed (ANOVA *p*-value < 0.0001) by Tukey’s post-test. *** *p* < 0.001 compared with sham control, ^##^ *p* < 0.01, ^###^ *p* < 0.001 compared with AA alone. OF.Cr = *Otostegia fruticosa* crude extract. AA = acetic acid; PO = orally; IR = intrarectally.

The colon of the colitis animals exhibited a flabby exterior, bowel wall thickening, and ulceration. All animals were assessed for ulcer lesion score and ulcer area. Ulcer index (UI) was calculated as the sum of lesion score and ulcer area. The UI was found to be significantly increased in colitis rats; however, pretreatment with OF.Cr at both the doses (200 and 400 mg/kg) and prednisolone expressively resulted in decreased UI. The observed results supported the idea that OF.Cr diminished colonic injury (Figure 5).

### 3.3. Effect of O. fruticosa Extract on Lipid Peroxidation

**Figure 6:**
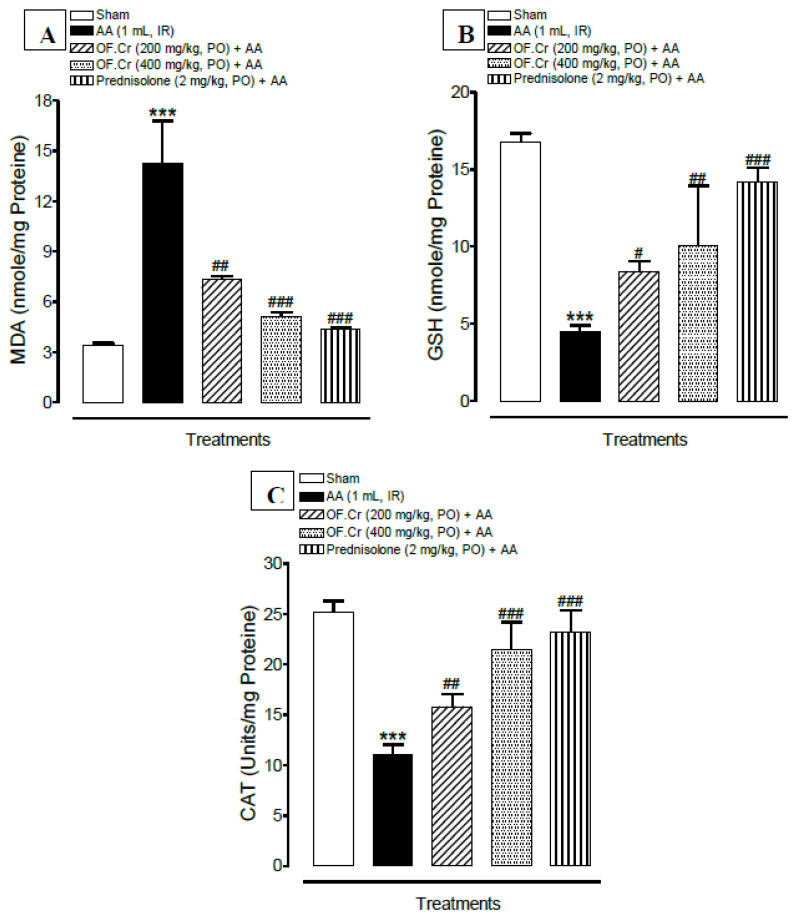
Effect of *Otostegia fruticosa* on (**A**) malondialdehyde (MDA), (**B**) total glutathione (GSH), and (**C**) catalase (CAT) in AA-induced colitis rats. Data expressed as median (IQR) (n = 6). One way ANOVA was used for statistical analysis (ANOVA P-value <0.0001) followed by Tukey’s post-test. *** *p* < 0.001 compared with sham control, ^#^ *p* < 0.05, ^##^ *p* < 0.01, ^###^ *p* < 0.001 compared with AA alone. OF.Cr = *Otostegia fruticosa* crude extract. AA = acetic acid; PO = orally; IR = intrarectally.

Figure 6A depicts the effect of OF.Cr on lipid peroxidation in the colon tissue of all animals. AA administration significantly (*p* < 0.001) increased the mean MDA level compared to sham rats. Animals treated with prednisolone and OF.Cr at 200 and 400 mg/kg exhibited a significant decrease in mean MDA levels compared to colitis animals.

### 3.4. Effect of O. fruticosa Extract on Antioxidant Activity

Figure 6 (B and C) depicts the effect of OF.Cr on antioxidant activity in the PMS of the colon tissue of all animals. AA-induced colitis animals exhibited significantly reduced levels of catalase (CAT) and total glutathione (GSH), however, anti-oxidant levels were consistently increased in the treatment groups compared to colitis animals.

The GSH level was found to be reduced significantly (*p* < 0.001) in colitis rats when compared with the sham control. OF.Cr at both doses of 200 and 400 mg/kg and prednisolone exhibited significantly increased levels of GSH compared to colitis rats (Figure 6B).

Figure 6C depicts decreased CAT activity in colitis rats compared to the sham control. However, OF.Cr pretreatment at doses of 200 and 400 mg/kg resulted in significantly enhanced activity of CAT.

### 3.5. Effect of O. fruticosa Extract on IL-6 and TNFα

**Figure 7:**
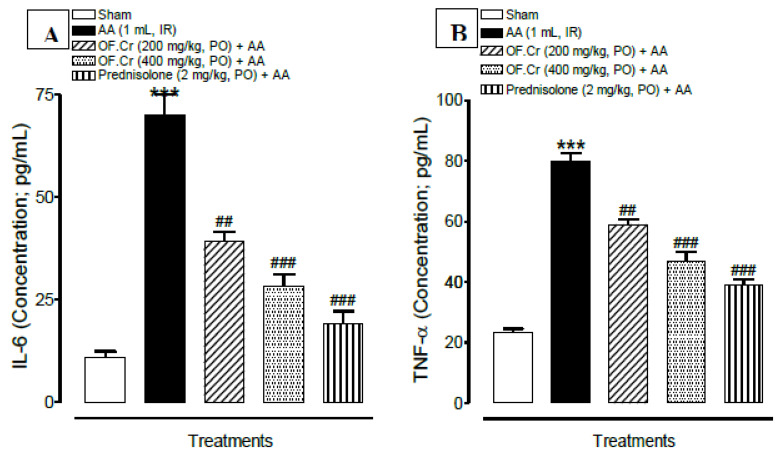
Effect of *Otostegia fruticosa* on (**A**) interleukin-6 (IL-6) and (**B**) tumor necrosis factor (TNF-α) and in AA-induced colitis rats. Data expressed as median (IQR) (n = 6). One way ANOVA was used for statistical analysis (ANOVA *p*-value < 0.0001) followed by Tukey’s post-test. *** *p* < 0.001 compared with sham control, ^##^ *p* < 0.01, ^###^ *p* < 0.001 compared with AA alone. OF.Cr = *Otostegia fruticosa* crude extract. AA = acetic acid; PO = orally; IR = intrarectally.

IL-6 plays a key role in UC. It has been reported earlier that inhibiting the IL-6 signal pathway could reduce the many pro-inflammatory factors and improve the colitis. The observed results exhibited significantly (*p* < 0.001) increased concentrations of IL-6 in colitis rats. Prednisolone significantly (*p* < 0.001) reduced this elevated concentration. OF.Cr pretreated rats also showed a significant (*p* < 0.001) decrease in the concentrations of IL-6 at doses of 200 and 400 mg/kg (Figure 7A).

The TNF-α concentration (pg/mL) was found to be significantly (*p* < 0.001) increased in the AA-induced colitis group. Pretreatment of rats with prednisolone and OF.Cr at doses of 200 and 400 mg/kg resulted in significantly decreased TNF-α concentration, as shown in Figure 7B.

### 3.6. Effect of O. fruticosa Extract on Microscopic Damage

Pretreatment of rats orally with increasing doses of OF.Cr (200 and 400 mg/kg) and prednisolone significantly improved the histopathological parameters when observed at 300x in their colonic extracted tissues with post-exposure of AA-induced colitis.

**Figure 8:**
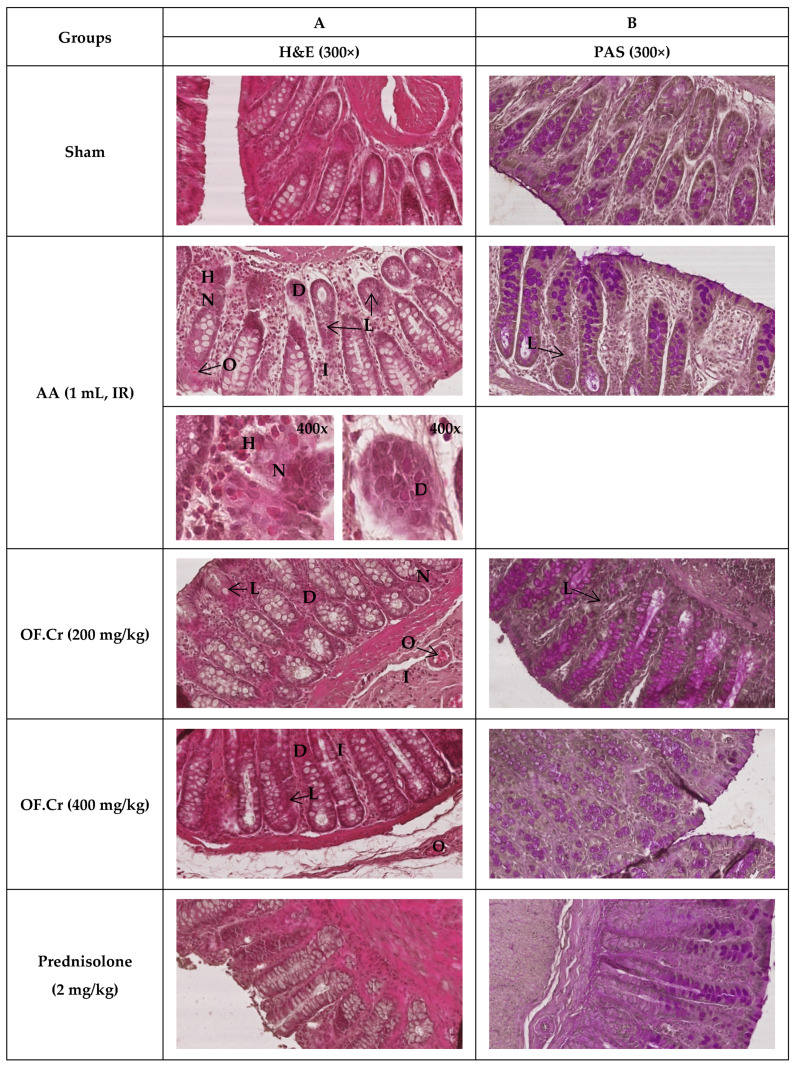
Effect of *Otostegia fruticosa* crude extract on histopathological changes in colon tissues in AA-induced colitis in Wistar albino rats (300×). Colon tissues were stained with (**A**) H&E and (**B**) PAS for microscopic evaluation. Sham control rats showing the normal morphological structure in both the stained photomicrographs. AA administered rats with H&E stain showing severe tissue damage of both mucosal and submucosal layers of intestine expressed as complete loss of goblet cells (L), remarkable necrosis (N), and degeneration (D), as well as occlusion (O) of blood vessels and infiltration (I) of mucosa and submucosa by inflammatory cells. PAS staining shows severe loss of mucin and goblet cells. Pretreated rats with low doses (200 mg/kg) of *O. fruticosa* crude extract show better improvement compare to high doses (400 mg/kg), whereas prednisolone showed complete protection against AA-induced colon damage. OF.Cr = *Otostegia fruticose* crude extract, AA = acetic acid, H&E = hematoxylin and eosin, PAS = periodic acid Schiff’s.

The photographs, as shown in Figure 8, represents the colon tissue sections extracted from respective groups of rats and were processed with general stain (H & E) for histological examination. No morphological alterations in the structure of the sham control colonic samples were observed, whereas the diseased group rat’s colon samples were found with severe damage areas of the mucosal and submucosal layers, infiltration of inflammatory cells, loss of most goblet cells, significant necrosis, and degeneration, as well as obstruction of arteries and veins. Rats pretreated with 200 mg/kg OF.Cr showed gross improvement in respective colonic tissues; however, moderate tissue damage was still present, as was observed in the form of tissue necrosis, degeneration, obstruction of blood vessels, and infiltration of mucosa and submucosa. They also showed much loss of goblet cells. The high dose showed better improvement but still suffering from low tissue damage.

The assessment of goblet cell depletion and type of mucin (neutral, acidic, basic) in the different studied groups of rats was conducted after staining the processed colonic samples with PAS. In the sham-treated group colonic samples, the appearance of normal goblet cells at the mucosal layer was seen. Colonic samples of the colitis group showed purple goblet cells, indicating the presence of acidic mucin in the area of the re-epithelized mucosa with evidence of marked mucin depletion. Pretreatment of OF.Cr and prednisolone not only protected the goblet cells from diminution but also retained the neutral mucin at the mucosal layers in the colon samples. Pretreated rats with OF.Cr exhibited dose-dependent improvement in protection against AA-induced colon damage.

Multiple scoring systems that gave different information were followed for quantifying histological damage and mucin contents in the colonic samples of the different studied groups. Neurath, Kiela, and Mucin scoring showed major damage in the AA group when compared with the sham group, whereas OF.Cr markedly decreased this damage similar to prednisolone, in a dose-dependent manner (Table 1). The increasing doses of OF.Cr, similar to prednisolone, cause retention of a significant number of goblet cells during mucin staining quantification conducted through the scoring system compared to AA group colonic samples that show non-significant staining, whereas the sham control showed 100% goblet cells stained.

## 4. Discussion

The leaf and stem part of *O. fruticosa* has been reported for its traditional use in the treatment of various disorders [22,24]. However, the effort to explore its traditional use for the treatment of colitis is still unknown. In the present study, the ameliorative effect of *O. fruticosa* crude extract (OF.Cr) was investigated against AA-induced UC in Wistar albino rats.

The colitis induced in this study by intrarectal (IR) administration of 5% AA in Wistar albino rats exhibited signs comparable to colitis patients, such as diarrhea, blood in the stool, decreased body weight, and reduction in colon length, which corroborates with the previous studies [44]. The findings of the present study for the first time demonstrate that OF.Cr significantly diminishes the severity of colonic impairment induced by AA.

Weight loss is an indicator of the severity of intestinal inflammation and correlates with the histopathological changes of colitis [45]. It has been reported earlier that, in colitis, increased levels of cytokines such as TNF-α and IL-6 have a significant contribution to body weight reduction by the release of appetite suppressive neuropeptides [46]. Additionally, in the present study, AA administration resulted in significantly decreased body weight, which might be due to decreased food intake or malabsorption, and massive fluid loss through rectal bleeding and diarrhea.

The extent of the AA-induced colitis could be evaluated by the DAI score. Colon length is inversely related to the severity of inflammation of the colon. The observed findings showed an increased DAI score, histopathological score, and shortening of colon length in AA-induced colitis rats. The observed increased colon and spleen weight/length ratio in the UC animals might be due to severe edema, necrosis, and inflammatory cell infiltration [19,47]. However, pre-treatment of colitis rats with oral administration of OF.Cr at both doses (200 and 400 mg/kg) resulted in a significant decrease in the DAI score, and restored the colon length. Treatment with OF.Cr significantly restores the histopathological deterioration of colon tissue, including mucosal injury, necrosis, degeneration, obstruction of arteries and veins, and infiltration of inflammatory cells, as compared to colitis rats. The ameliorative effect of OF.Cr was comparable to prednisolone.

One of the main causative factors of inflammatory diseases is oxidative stress and suppressed antioxidant defense systems that regulate the production of reactive oxygen species (ROS) [48]. Consequently, it resulted in overproduction of ROS and decreased enzymatic (CAT) and non-enzymatic (GSH) antioxidants, which initiates a reactive oxygen metabolites (ROM) cascade and causes lipid peroxidation (LPO) [49]. In the present study, increased oxidative stress was observed in AA-induced colitis rats, which was confirmed by increased MDA levels as well as decreased non-enzymatic antioxidant (GSH) levels and enzymatic antioxidant (CAT) activity in colon tissues. The findings from the present study confirmed that OF.Cr was capable to protect mucosal damage by significantly reducing the MDA levels and promoting the GSH and CAT levels in the colon tissues of colitis rats. Our results corroborate the findings of earlier studies [50].

To further evaluate the mechanisms involved in the protective effect of OF.Cr in colitis, variables were designated, as these were significant to the colitis model. AA causes colonic epithelium damage, which results in the access of acid leading to widespread intracellular acidification [51]. Ultimately, it results in the activation of monocytes and macrophages, which are well-known for releasing IL-6 and TNF-α. These cytokines (IL-6 and TNF-α) further cause the release of various chemokine and release effective cytotoxic oxidants that promote chemotaxis [52]. TNF-α is mainly consider to be involved in increased endothelia cell permeability, decreased pain threshold, leukocyte generation, and increased prostaglandins (PGs) levels. TNF-α is reported to be elevated in the blood, stool samples, and mucosa [53,54] of UC patients. These findings, together with the effectiveness of anti-TNF treatment for UC, corroborate the importance of TNF-α in the pathogenesis of the disease. On the other hand, IL-6 stimulates the acute-phase proteins synthesis and complement system activation [55]. UC is a deteriorating inflammatory disorder characterized by an imbalanced intestinal mucosal immune system that includes dysregulated immune response and an imbalance of pro-inflammatory cytokines (TNF-α, and IL-6) release. Therefore, promising regulation of these pro-inflammatory cytokines could be a realistic approach for the treatment of UC. In the present study, OF.Cr treatment resulted in significantly decreased release of TNF-α and IL-6, as compared with the AA-induced colitis rats.

Previous studies reported that disturbed epithelial barrier is a preliminary incident in IBD that results in the expression of the disease [56]. It was reported earlier that the AA-induced colitis model also showed comparable incidence by initiating epithelial injury and decreased goblet cells, therefore resulting in decreased permeability of the colon and increased bacterial translocation to the colon [52,57]. A comparable pattern of damage was also reported in the present study as the colon sections of AA-induced colitis group stained with PAS stain displayed deceptively decreased goblet cells and reduced mucin score. However, this reduction was significantly restored in the OF.Cr pretreated rats, confirming reduced colon permeability. Further assessment with general (H&E) staining exhibited the loss of epithelial lining and infiltration of inflammatory cells in colitis rats, which corroborates the earlier studies [58]. Hence, the observed mucosal protective effect of OF.Cr might be due to the fact that it reduces goblet cell diminution and neutrophil infiltration.

In a previous study, the OF.Cr analyzed by GC-MS showed the presence of terpenes, terpene alcohols, aliphatic hydrocarbons, higher fatty acids, and sterols [26]. Santalene, Lavandulyl acetate, Solanesol, and Salvialene were found to be the major component of OF.Cr.

Sharma et al. [59] reported that Sandalwood oil, and its primary constituents, α- and β-santalols, can suppress LPS-mediated pro-inflammatory responses by inhibiting cyclooxygenase activity and cytokine/chemokine expression in skin models. Among eight sesquiterpenic hydrocarbons found in the petroleum ether fraction, β-santalene was reported to have the highest anti-inflammatory activity [60]. According to previous studies, in addition to curcumin, other constituents of turmeric, primarily essential turmeric oils comprising of aromatic-turmerones (ar-turmerones), α-turmerones, β-turmerones, α-santalene, and aromatic curcumene, also possess significant anti-inflammatory and anti-oxidant properties [61,62]. Solanesol possesses strong free radical absorption ability and anti-inflammatory and antioxidant activity [63]. The observed biological activities of OF.Cr might be because of the presence of terpenes, Santalene, Lavandulyl acetate, Solanesol, and Salvialene [64].

The acetic acid-induced colitis model is one of the limitations of our current study, and we plan to test the protective effect of OF.Cr in the dextran sodium sulphate (DDS)-induced ulcerative colitis model, which is considered more sophisticated and will extract more information about the innate immunity. Another limitation of the present study is detailed chemical characterization and identification of the main active ingredients involved in the protective effect of this plant against UC, which will be addressed in our future studies. Additionally, in future studies, we plan to perform histopathological analysis by more than one observer, which is a limitation of the present study.

## 5. Conclusions

As per the literature survey, the present study is an initial attempt to investigate the ameliorative effect of OF.Cr against the AA-induced model of UC. The findings highlight that *Otostegia fruticosa* protects the severity and extent of colitis in rats by controlling the release of TNF-α and IL-6 as well as the antioxidant effect that resulted in mucosal protective effects. Hence, the results presented herein indicate that *Otostegia fruticosa*, by targeting multiple features of the disease, has the potential to treat UC and therefore, in the future, hopefully it could be developed as a candidate for ulcerative colitis.

## Figures and Tables

**Table 1:** Quantification of microscopic damage.

Groups	Neurath Score (Maximum Score = 4)	Kiela Score (Maximum Score = 3)	Mucin Score (Maximum Score = 3)
AA	3.75	2.75	1.25
OF.Cr (200 mg/kg)	2.75	2.25	2.00
OF.Cr (400 mg/kg)	1.75 **	2.00 *	2.00
Prednisolone (2 mg/kg)	1.25 ***	1.00 ***	2.75 ***
KW p value	0.0004	0.0007	0.0029

Data are expressed as median. Results for the OF.Cr treatment group were compared against the AA group with Kruskal–Wallis test followed by Dunn’s Multiple Comparisons Test. * *p* < 0.05 vs. AA; ** *p* < 0.01 vs. AA; *** *p* < 0.001 vs. AA. OF.Cr = *Otostegia fruticose* crude extract; AA = acetic acid.

## Data Availability

Data sharing not applicable.

## Supplementary Materials

The following are available online at https://www.mdpi.com/2075-1729/11/3/195/s1, Table S1: Scoring of the disease activity index.
