# Supplementary material for: Role of Oxidative Stress and Inflammatory Cytokines (TNF-α and IL-6) in Acetic Acid-Induced Ulcerative Colitis in Rats: Ameliorated by Otostegia fruticosa
Source: Life (Basel). 2021 Mar 3;11(3):195. doi: 10.3390/life11030195 (PMC8001148; doi:10.3390/life11030195)
Supplement: Supplementary file 1 [file life-11-00195-s001.pdf]

Table S1: Scoring of the disease activity index.

Weight loss (%)	Stool consistency	Occult/gross bleeding	Score
Normal	Normal	Normal	0
1-5	Soft but still formed		1
5-10	Loose stools	Hemo-occult positive	2
10-20	Diarrhea		3
>20		Gross bleeding	4

Note. Normal stools = well-formed pellets, loose stools = pasty and semi-formed stools which do not stick to the anus and diarrhea = liquid stools that stick to the anus.
